# Characterization of Ultrasonic Vocalization-Modulated Neurons in Rat Motor Cortex Based on Their Activity Modulation and Axonal Projection to the Periaqueductal Gray

**DOI:** 10.1523/ENEURO.0452-23.2024

**Published:** 2024-03-29

**Authors:** Aamir Sharif, Jumpei Matsumoto, Chinzorig Choijiljav, Amarbayasgalant Badarch, Tsuyoshi Setogawa, Hisao Nishijo, Hiroshi Nishimaru

**Affiliations:** ^1^Department of System Emotional Science, Faculty of Medicine, University of Toyama, Toyama 930-0194, Japan; ^2^Research Center for Idling Brain Science, University of Toyama, Toyama 930-0194, Japan; ^3^Department of Sport and Health Sciences, Faculty of Human Sciences, University of East Asia, Shimonoseki 751-0807, Japan

**Keywords:** motor cortex, optogenetic tagging, periaqueductal gray, rat, single unit recording, ultrasonic vocalizations

## Abstract

Vocalization, a means of social communication, is prevalent among many species, including humans. Both rats and mice use ultrasonic vocalizations (USVs) in various social contexts and affective states. The motor cortex is hypothesized to be involved in precisely controlling USVs through connections with critical regions of the brain for vocalization, such as the periaqueductal gray matter (PAG). However, it is unclear how neurons in the motor cortex are modulated during USVs. Moreover, the relationship between USV modulation of neurons and anatomical connections from the motor cortex to PAG is also not clearly understood. In this study, we first characterized the activity patterns of neurons in the primary and secondary motor cortices during emission of USVs in rats using large-scale electrophysiological recordings. We also examined the axonal projection of the motor cortex to PAG using retrograde labeling and identified two clusters of PAG-projecting neurons in the anterior and posterior parts of the motor cortex. The neural activity patterns around the emission of USVs differed between the anterior and posterior regions, which were divided based on the distribution of PAG-projecting neurons in the motor cortex. Furthermore, using optogenetic tagging, we recorded the USV modulation of PAG-projecting neurons in the posterior part of the motor cortex and found that they showed predominantly sustained excitatory responses during USVs. These results contribute to our understanding of the involvement of the motor cortex in the generation of USV at the neuronal and circuit levels.

## Significance Statement

Ultrasonic vocalizations (USVs) in rodents have been widely used as experimental models to study neural mechanisms and deficits in social, emotional, and motor functions in mammals. However, the involvement of the motor cortex has not yet been fully characterized. In this study, we investigated the neural activity around USVs across the rat motor cortex and its relationship with projections to the PAG, which plays a central role in producing USV. The results demonstrated strong involvement of the motor cortex in USVs at both neuronal and circuit levels. This study provides a basis for future studies examining the cortical control of USVs using genetic and physiological manipulations.

## Introduction

Laboratory rats and mice communicate with each other using ultrasonic vocalizations (USVs), which are inaudible to humans, in response to various social contexts and affective states (Portfors, 2007). USVs have been widely used as experimental models to study neural mechanisms and deficits in social, emotional, and motor functions in mammals (Konopka and Roberts, 2016; Han et al., 2019; Simola and Granon, 2019; Karigo, 2022; Premoli et al., 2023). Understanding the neural mechanisms that control USVs provides an important basis for facilitating such studies.

Extensive studies have shown that subcortical regions, particularly the periaqueductal gray matter (PAG) in the midbrain, plays a central role in controlling mammalian vocalizations, including USVs, in rodents (Gruber-Dujardin, 2010). Recent studies in mice and rats have shown that the activation of PAG elicits USVs and that its inhibition blocks USVs (Tschida et al., 2019; Gloveli et al., 2023). PAG directly projects to brainstem regions involved in vocal–respiratory pattern generation (Gruber-Dujardin, 2010; Tschida et al., 2019). Other subcortical areas, including the preoptic area of the hypothalamus and the amygdala, have been reported to control USVs by activating the PAG depending on the social context (Michael et al., 2020; Chen et al., 2021; Xiao et al., 2023). However, only a few studies have investigated the role of cortical areas, including the motor cortex, in controlling USVs. This may be partly due to the disputed role of cortical control in USVs, particularly in mice. A previous study showed that seemingly normal USVs were observed in mice lacking majority of the cortex, suggesting that cortical structures may not be critical for controlling USVs (Hammerschmidt et al., 2015). However, several anatomical tracing and immediate-early gene expression studies in mice have shown that the motor cortex projects to the PAG and nucleus ambiguus, a brainstem nucleus controlling the vocal muscles, and that it has a reciprocal connection with the auditory cortex (Arriaga et al., 2012; Nelson et al., 2013). Moreover, lesions of the motor cortex reportedly increase the standard deviation of pitch distribution, which also occurs after hearing loss (Arriaga et al., 2012). Furthermore, a recent deep neural network study in mice lacking majority of the cortex showed alteration of USVs in comparison with littermate control mice (Ivanenko et al., 2020). These results suggest that the motor cortex is involved in precise controlling of USVs, such as maintenance of quality of USVs based on auditory feedback. However, it remains unclear how the neurons distributed across the motor cortex respond dynamically to USVs.

Herein, we report the neural activity around emission of USVs across the primary (M1) and secondary motor cortices (M2) in rats using large-scale electrophysiological recordings. We found that the neural activity patterns around emission of USVs differed between the anterior and posterior parts of the motor cortex, in parallel with the differences in their anatomical projections onto the PAG. We also found that the responses of PAG-projecting neurons in the posterior motor cortex were predominantly excitatory.

## Materials and Methods

### Animals

A total of 26 adult male Wistar rats (weighing 320–530 g; Jackson Laboratory) and 11 adult female Wistar rats (weighing 200–300 g; Japan SLC) were used in this study. Male rats were used as experimental subjects and females were used as stimuli to induce vocalization in the subjects, since this situation has been reported to induce vocalizations in rats effectively (Brudzynski, 2009). The rats were housed as two or three animals per cage. Rats with implanted electrodes were kept individually to avoid the risk of damage to the implant by cage mates. All animals were housed in temperature-controlled rooms with a standard 12 h light/dark cycle and had *ad libitum* access to food and water. All animal procedures were performed in accordance with the University of Toyama animal care committee's regulations.

### Surgery

Subject male rats were anesthetized with an intraperitoneal injection of a mixture of medetomidine (0.375 mg/kg), midazolam (2.0 mg/kg), and butorphanol (2.5 mg/kg) or using a combination of intraperitoneal sodium pentobarbital (15 mg/kg) and isoflurane gas (2%). To record neural signals, a custom microdrive array (Kloosterman et al., 2009) including 16 independently adjustable tetrodes (twisted bundles of four 12.7 μm polyimide-coated nichrome wires, gold plated to an impedance of ∼300 kΩ; Sandvik) was implanted in the left cortical areas including the primary and secondary motor cortex (M1 and M2; AP, −2.0 to 4.5 mm; ML, 0.5 to 4.5 mm). For optogenetic tagging of recorded neurons, in some rats, five optic fibers were implanted above the tetrodes (0.2 mm from cortical surface), and AAV-retro-hSyn-hChR2-EYFP (0.1 μl; 26973-AAVrg, Addgene) was injected into the left PAG (AP, −7.8 mm; ML, 0.7 mm; DV, 5.6 mm; Paxinos and Watson, 2007) using a motorized stereotaxic microinjector (IMS-20; Narishige) through a pipette angled 15° to the lateral. In a separate group of male rats, to label neurons projecting to the PAG, AAV-retro-hSyn-EGFP (0.1 μl; 50465-AAVrg, Addgene) was similarly injected to the left PAG. Stimulus adult female rats were ovariectomized under anesthesia with an intraperitoneal injection of the mixture described above.

### Recording neural activity during USV emission

For recording, the subject male rats were first placed in an elevated recording arena (25 × 25 cm; height, 0.9 m). Then, two different stimulus females were alternately placed in the recording arena shortly (1–3 min duration) for four times to induce USVs, with 3–4 min intervals without females in the arena. The subject male rat often continued to vocalize during this interval. On most of the recording days, to maintain the motivation of the subjects to vocalize, one or two female rats were brought to the estrous phase by subcutaneously injecting estradiol benzoate (5 μg/rat) and progesterone (500 μg/rat) 48 h and 4–7 h, respectively, before recording. The neural signals were amplified and digitized at 30 kHz sampling rate using an Open Ephys system (Open Ephys Acquisition Board + Open Ephys 32-ch headstage with 3-axis accelerometer). Videos were captured at 30 frames/s using four RGBD cameras (Realsense R200, Intel) surrounding the arena. USVs were recorded and digitized at 768 kHz, using an ultrasonic microphone (CMPA/CM16, Avisoft), a custom amplifier, and an analog-digital converter (Katou Acoustics Consultant Office). The neural, video, and audio recordings were synchronized using the timestamp signals of the video frames sent to the neural and audio data acquisition system.

After the behavioral session described above, each optical fiber was connected to a head-mounted blue LED module (Plexon) for optogenetic tagging. Then, trains of 10 light pulses (pulse duration, 10 ms; light power, 10 mW max; frequency, 5 Hz) were applied 10 times with 10 s intertrain intervals.

After each recording day, the tetrodes were lowered by at least 75 μm to record new neurons on the following recording day.

### Histology

**Figure 1.**
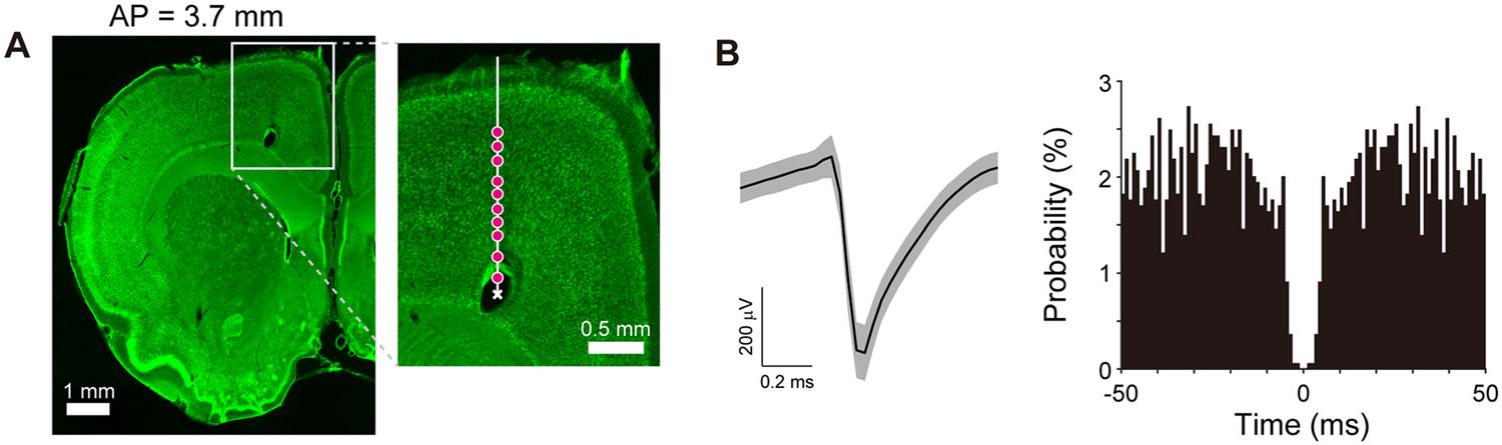
Representative examples of electrophysiological recording sites and unit waveform. ***A***, An example of a coronal section (Nissl stained) with an electric lesion at the final location of the electrode (indicated by a white cross). Red circles indicate the recording sites of neurons estimated from the final position and microdrive rotation. ***B***, Left panel, An example of the spike waveforms of a motor cortex neuron (in M2). Shaded area represents mean ± SD. Right panel, Autocorrelogram of the neuron shown on the left with a bin of 1 ms.

After electrophysiological recording, rats were deeply anesthetized with intraperitoneal injection of the anesthetic mixture of medetomidine (0.75 mg/kg), midazolam (4.0 mg/kg), and butorphanol (5.0 mg/kg), and the recording sites were marked using electrolytic lesions by passing a 40 μA negative current through the recording electrodes for 10 s. The rats were then perfused through the heart with 0.9% saline, followed by 4% paraformaldehyde. Brain was removed, postfixed overnight, and placed in 15, 25, and 30% sucrose solutions, respectively, until the brain sank to the bottom of the container of 30% sucrose solution. Postfixation sucrose replacement took a total of 6–8 d. For recording site verification, the brains were sectioned at a thickness of 50 or 100 μm. For counting neurons expressing fluorescent markers, rats were perfused 4 weeks after AAV injection, and 50 μm brain sections were made in the same way described above. Sections were stained with fluorescent Nissl (NeuroTrace, Thermo Fisher Scientific), mounted using Fluoromount-G mounting medium (Southern Biotech), and visualized under a fluorescence microscope. The recording sites of the neurons were reconstructed from the final position of the tetrodes and rotation of the microdrives according to the corresponding sections of the rat brain atlas (Paxinos and Watson, 2007; Fig. 1*A*).

### Data analysis

#### Spike sorting

Offline spike-sorting was performed using KiloSort (an automatic spike sorting software; Pachitariu et al., 2016) followed by manual curation using Klusters (Hazan et al., 2006). Each isolated cluster of neuronal spikes was then manually assessed to ensure that the waveform shapes were consistent with the action potentials (Fig. 1*B*). In addition, an autocorrelogram was constructed for each isolated cluster. An absolute refractory period of at least 1.0 ms was used to exclude suspected multiple units (Matsumoto et al., 2016). Units with <100 recorded spikes were excluded as it was difficult to determine their quality. Spike width (trough-to-peak duration) of averaged spike waveform for each unit was calculated, and neurons were accordingly classified into either wide or narrow spiking neurons which corresponded to putative pyramidal neurons or putative interneurons, respectively (Barthó et al., 2004).

#### USV segmentation

USV syllables in the audio recordings were detected using USVSEG (Tachibana et al., 2020) with the following parameters: frequency range, 20–120 kHz; minimum syllable duration, 5 ms; and minimum gap between syllables, 20 ms. Manual curation was performed to exclude false-positive detections (detecting noise as USV) and inaccurate segmentations (e.g., noise segmented with actual syllables).

#### Classification of neurons responding to USVs

**Figure 2.**
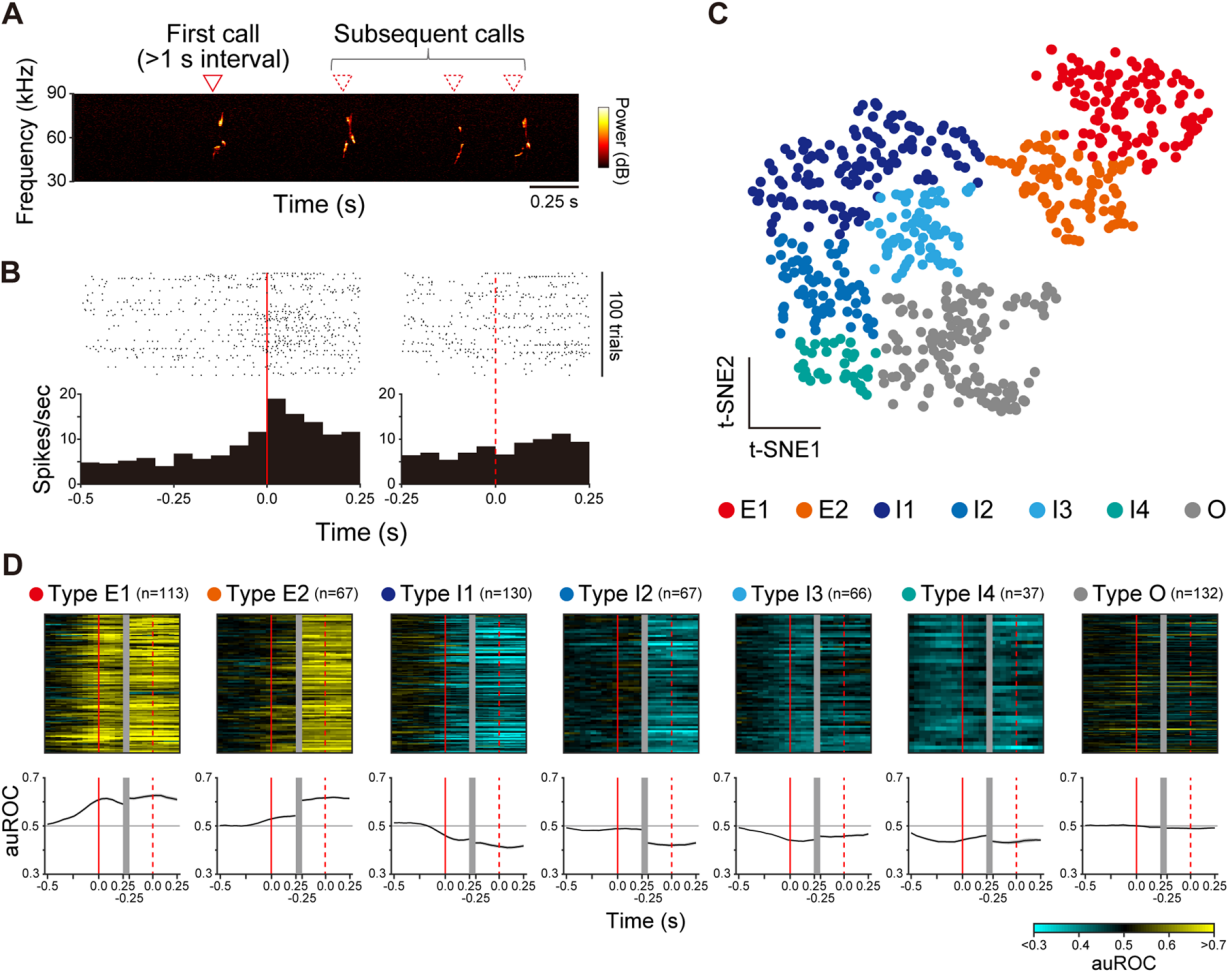
Classification of motor cortex neurons responding to USVs. ***A***, An example spectrogram of a USV bout. ***B***, Neural response of a motor cortex neuron (in M1) around USVs shown by raster displays and their summed histograms; red solid and dotted lines represent the onset of first and subsequent calls, respectively; bin width of the histogram, 50 ms. ***C***, USV-responsive neurons mapped into two-dimensional plane using t-SNE based on their response features. Each point color indicates the response type of the neuron, classified using the watershed transform algorithm (Extended Data Fig. 2-2). ***D***, Response patterns of all the neurons classified into each type (top) and their average (bottom). Supplementary information to this figure can be found in Extended Data Figures 2-1 to 2-3.

In the following analysis, to prevent potential contamination of the stimulus female's USVs, we focused on analyzing neural activity around USVs during the interval in which the stimulus female rat was absent from the recording arena. USV syllables were separated into first calls (syllables with >1 s intervals from the previous syllables) and subsequent calls (others; Fig. 2*A*). Significant neuronal responses to USVs were determined using Wilcoxon rank sum test with Bonferroni’s corrections (*p* < 0.05, repetitions, 4) comparing a baseline firing rate (−1.0 to −0.5 s from the first call onset) with 0.5 s before and 0.25 s after the first call onsets and 0.25 s before and after the subsequent call onsets. The response patterns of responsive neurons were further classified as follows. Mean firing rates of each 50 ms bins in 1.0 s before and after the onset of the first and subsequent calls were calculated (Fig. 2*B*) and smoothed by a moving average (window size, 5 bins). Receiver operating characteristic (ROC) curves were calculated by comparing the distribution of firing rates across trials in 50 ms bins to the distribution of the baseline firing rates, and the area under the ROC curve (auROC) at each time bin was calculated (Cohen et al., 2012; Kremer et al., 2020). A 25-dimensional response pattern vector was constructed with auROC values of −0.5 to 0.25 s around the first call onsets and −0.25 to 0.25 s around the subsequent call onsets, and then principal component analysis (PCA) was performed with the vectors of all responsive neurons. The resultant first three principal components (PC 1–3) and common logarithm of baseline firing rate were used as features for the following unsupervised classification, according to a previous study (Berman et al., 2014). First, the *z*-scores of four-dimensional features of the responsive neurons were mapped onto a two-dimensional plane using *t*-distributed stochastic neighbor embedding (t-SNE; Extended Data Fig. 2-2*A*). A watershed transform was then applied to a Gaussian-smoothed density of neurons to segment the space (Extended Data Fig. 2-2*B*,*C*). Finally, the neurons were classified based on their segmentation (Fig. 2*B*).

10.1523/ENEURO.0452-23.2024.f2-1 Figure 2-1 **Number of recorded neurons across the motor cortex.** Number of neurons recorded around each AP and ML coordinate is shown in the bubble chart. Shaded areas correspond to M1 and M2 on the brain surface, plotted according to the rat brain atlas (Paxinos and Watson, 2007). Download Figure 2-1, TIF file.

10.1523/ENEURO.0452-23.2024.f2-2 Figure 2-2 **Unsupervised classification using watershed algorithm.** From the two-dimensional plot obtained using t-SNE (**A**), a 2D histogram was generated and smoothed using a Gaussian filter (**B**). Individual peaks were isolated via watershed transform (**C**). Download Figure 2-2, TIF file.

**Figure 3.**
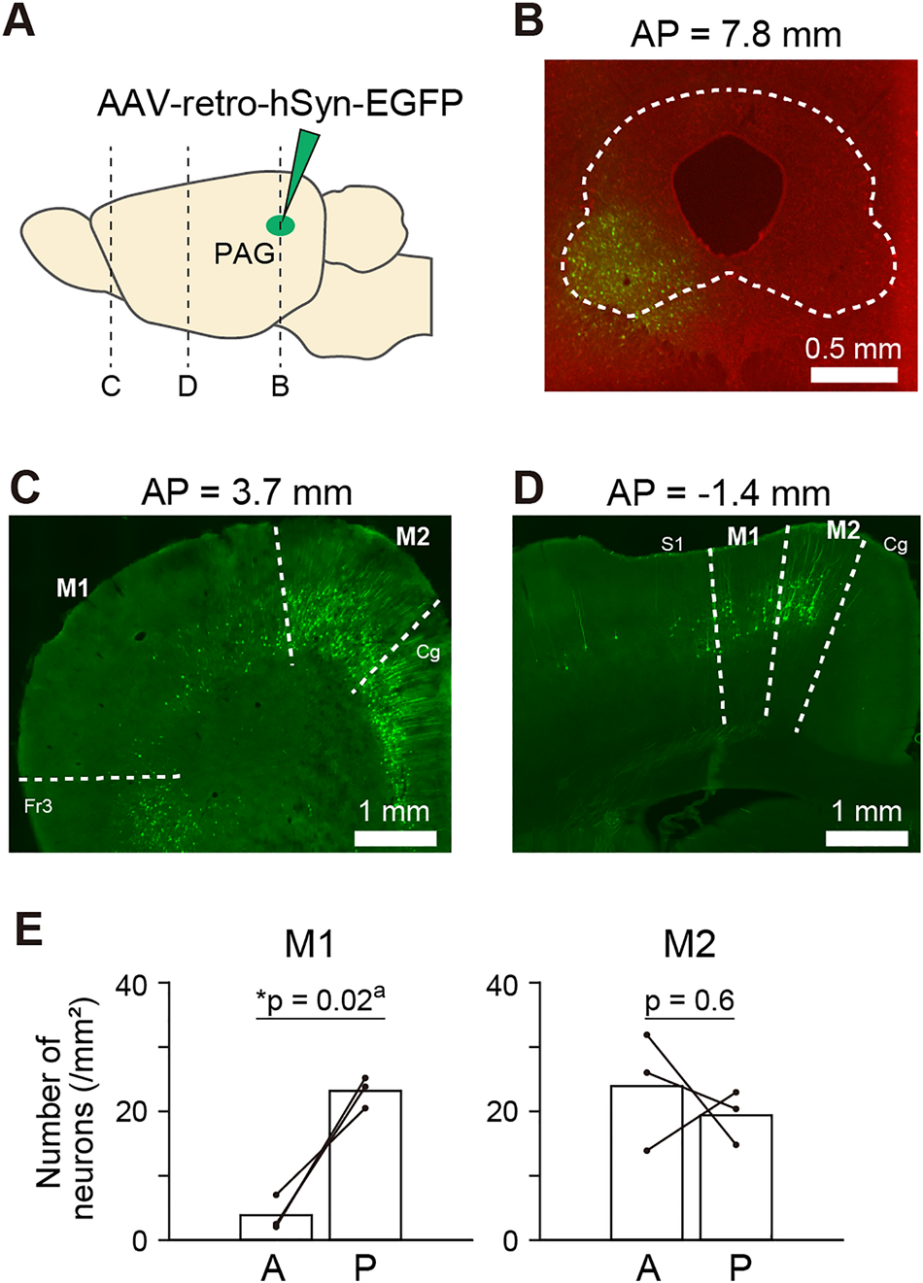
Distribution of PAG-projecting neurons in the motor cortex. ***A***, Schematic of AAV-retro-hSyn-EGFP injected into the PAG. The dotted lines indicate the locations of the coronal section images shown in panels ***B–D***. ***B***, A brain slice image showing the injection site. Red, Nissl. ***C***, ***D***, brain slice images of the anterior (***C***) and posterior (***D***) part of the motor cortex in the same brain sample shown in ***B***. Fr3, frontal cortex area 3; S1, primary somatosensory cortex; Cg, cingulate cortex. ***E***, Comparison of labeled cell densities in M1 (left) and M2 (right) between the anterior (A) and posterior (P) parts divided at AP = 1.5 mm. *p < 0.05, paired *t* test. ^a^Details of the statistical test is shown in Table 2. Supplementary information to this figure can be found in Extended Data Figure 3-1.

10.1523/ENEURO.0452-23.2024.f2-3 Figure 2-3 **Count of call-discriminating neurons.** **A**: Distribution of frequency ranges (max frequency - min frequency) of all recorded USVs (n = 20,195). The USVs were classified into flat (<15 kHz), and frequency modulated (FM; > 15 kHz) calls based on the values. **B**: Example spectrograms of the two call types. **C**: Percentage of call-discriminating neurons among each type of USV responsive neurons. Download Figure 2-3, TIF file.

To investigate the relationship between neural responses and call types, we classified USVs into two types based on their range of frequency, that is, flat (frequency range, <15 kHz) and frequency modulated (frequency range, >15 kHz) calls according to previous studies (Matsumoto et al., 2016; Simola and Granon, 2019). Then, significant discriminations of call types were determined using Wilcoxon rank sum test (*p* < 0.05) comparing firing rates around the two types of calls (−0.25 to 0.25 s around the first call onsets).

#### Counting neurons expressing fluorescent markers

Neurons labeled with enhanced green fluorescent protein (EGFP) were manually counted using ROI tools in the ImageJ/Fiji software. For each brain slice image (50 μm thickness, captured with a 10× objective on BZ-9000, Keyence), M1 and M2 regions were annotated according to corresponding atlas section in Paxinos and Watson (2007). Then, the number of labeled neurons in each region was counted. Finally, the labeled neuron density in each region was calculated by dividing the neuron count by the area of the region.

#### Optogenetic identification of PAG-projecting neurons

To identify the motor cortex neurons projecting to the PAG expressing channelrhodopsin (ChR2), we used the Stimulus-Associated spike Latency Test (SALT; Kvitsiani et al., 2013). The test determined whether light pulses significantly changed a neuron's spike timing by comparing the distribution of first spike latencies relative to the light pulse, assessed in a 10 ms window after light stimulation, to 10 ms epochs in the baseline period (−150 to 0 ms from the onset of light stimulation; see Kvitsiani et al., 2013 for details). The criteria for light-responsive neurons were light-induced increase in firing rate, a SALT *p* value of <0.01 and a Pearson's correlation coefficient (*r*) between spontaneous and light-evoked waveforms of >0.9 (Kvitsiani et al., 2013; Eshel et al., 2015).

### Statistics

Statistical tests were performed using R (The R Foundation) and DABEST software suite (Ho et al., 2019). The significance threshold was set to 0.05.

## Results

### Neuronal activity in the motor cortex during USVs

First, we performed electrophysiological recordings of motor cortex neurons during USV emission (*n* = 20 rats; Extended Data Fig. 2-1). To characterize the neural responses to USVs, we separately analyzed the neural activity around the first call (first syllable in a vocal bout) and subsequent calls (other syllables; Fig. 2*A*) since the intervals between syllables within a series of syllables were very short (averaged interval, 366 ± 64 ms, mean ± SD; *n* = 114 sessions). Figure 2*B* shows an example of an excitatory-responsive neuron to USVs. Of the 2,026 recorded neurons, 632 responded significantly to the USVs. We classified the response patterns of these USV-responsive neurons by response normalization using auROC (Cohen et al., 2012; Kremer et al., 2020; see Materials and Methods) followed by unsupervised clustering using t-SNE and watershed algorithm (Berman et al., 2014; Fig. 2*C*; Extended Data Fig. 2-2; see Materials and Methods). The responsive neurons were classified into seven types (Fig. 2*D*): two excitatory responding types (Type E1, E2), four inhibitory responding types (Types I1–4), and other responsive neurons that showed weak and variable responses (Type O). Among 632 responsive neurons, 48 (7.6%) neurons differentially responded depending on call types (Extended Data Fig. 2-3). These results indicate that the activity of neurons in the motor cortex represents vocalization and that these neurons can be classified based on how their activity is modulated around the time of vocal emission.

### PAG projection and USV response differed between the anterior and posterior parts of the motor cortex

Different body parts are represented by different anatomical areas in the motor cortex (Neafsey et al., 1986). Therefore, we investigated whether similar functional localization/heterogeneity exists in USVs. To address this question anatomically, we investigated the distribution of motor cortex neurons projecting to the ventral and lateral PAG, which have been found to be important for USVs in previous studies (Tschida et al., 2019; Gloveli et al., 2023), by injecting retrograde adeno-associated virus (AAV) encoding a fluorescent protein into the corresponding area of the PAG (Fig. 3*A*,*B*; *n* = 3 rats). By examining the motor cortex, we found a heterogeneous distribution of labeled neurons. More number of labeled neurons were found in the posterior than in the anterior part of M1 (Fig. 3*C*, Extended Data Fig. 3-1), whereas labeled neurons were equally distributed in the corresponding anterior and posterior parts of M2 (Fig. 3*D*, Extended Data Fig. 3-1). Interestingly, in the anterior part, a cluster of labeled neurons was observed continuously from the M2 to the cingulate, prelimbic, and infralimbic cortices while much fewer labeled neurons were found in M1 (Fig. 3*C*, Extended Data Fig. 3-1). In contrast, the cluster of labeled neurons appeared to be more localized in the posterior part of the motor cortex (Fig. 3*D,E*, Extended Data Fig. 3-1). These results suggest that clusters localized in the anterior and posterior parts of the motor cortex may play different roles with the PAG.

**Figure 4.**
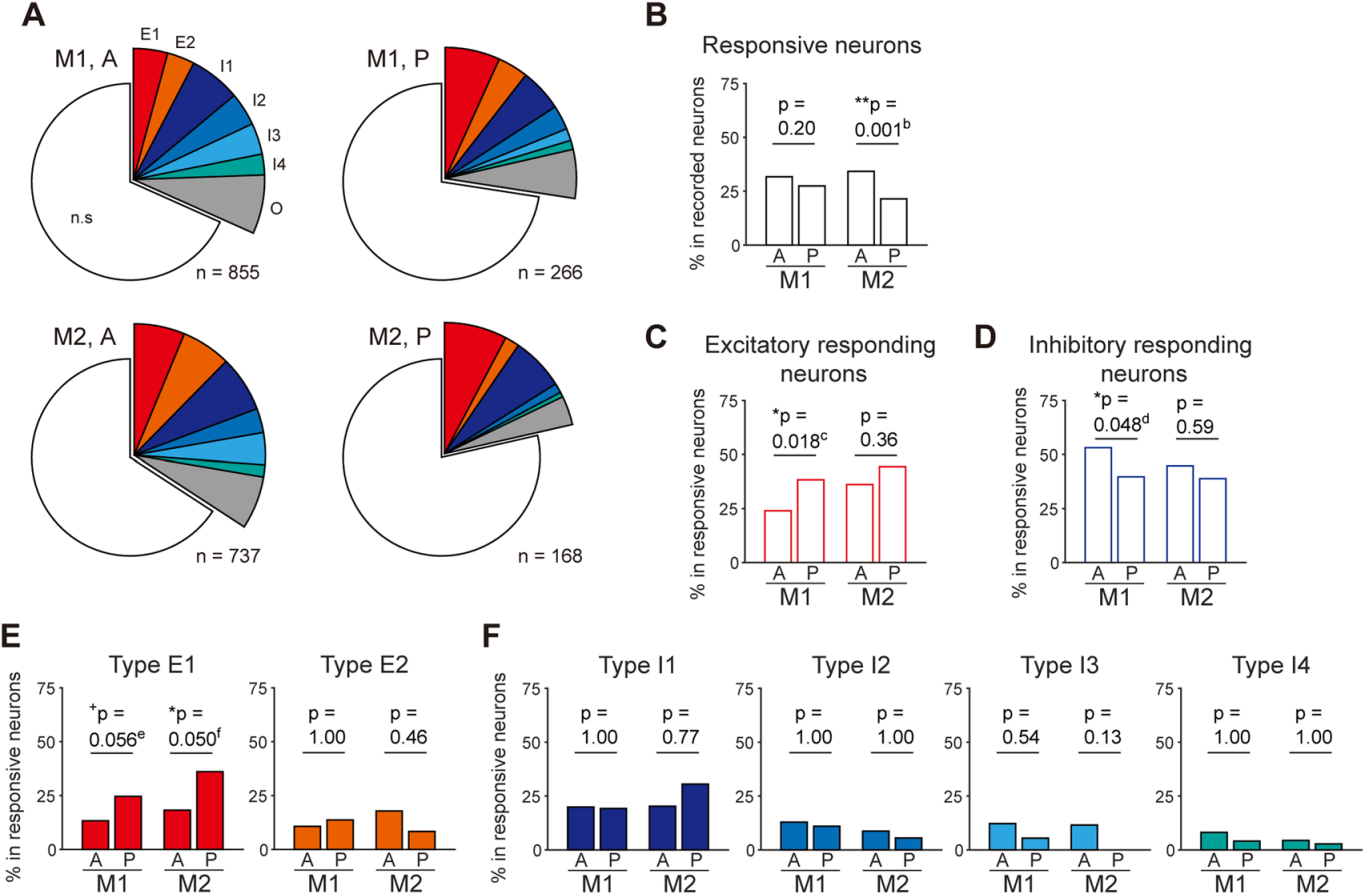
Proportion of different types of USV-responsive neurons in the anterior and posterior part of the motor cortex. ***A***, Pie charts showing the ratios of different types of USV-responsive neurons in the anterior (A) and posterior (P) parts of M1 and M2. ***B***, Percentage of responsive neurons among recorded neurons in each area. **p < 0.01, Fisher's exact test. ***C***, ***D***, Percentage of excitatory (Types E1 and E2; ***C***) and inhibitory (Types I1–4; ***D***) responding neurons among all responsive neurons. *p < 0.05, Fisher's exact test. ***E***, Percentage of each type of excitatory responding neuron among all responsive neurons. *p < 0.05, ^+^*p* < 0.1, Fisher's exact test with Bonferroni’s correction (repetition, 2). ***F***, Percentage of each type of excitatory responding neuron among all responsive neurons. Fisher's exact tests with Bonferroni’s correction (repetition, 4) showed no significant differences between the anterior and posterior parts. The corresponding numbers of neurons for each type in each area are listed in Table 1. ^b–f^Details of the statistical tests are shown in Table 2. Supplementary information to this figure can be found in Extended Data Figure 4-1. ^g–i^Details of the statistical tests are shown in Table 2.

10.1523/ENEURO.0452-23.2024.f3-1 Figure 3-1 **Distribution of the retrogradely-labeled cells in AP axis.** Densities at different AP levels for each animal are shown as single lines (blue, M1; red, M2). We divided the anterior and posterior part by 1.5  mm (dotted line) for the analyses in Fig. 3 and 4. Download Figure 3-1, TIF file.

Next, we examined whether the neuronal responses to USVs differed between the anterior and posterior parts of the motor cortex by comparing the number of different types of USV-responsive neurons between the regions (Fig. 4, Table 1). The total number of responsive neurons among the recorded ones tended to be higher in the anterior part than that in the posterior part (Fig. 4*B*). However, the proportion of excitatory responding neurons among all responsive ones was larger in the posterior part of M1 (Fig. 4*C*), whereas that of inhibitory responding neurons was larger in the anterior part of M1 (Fig. 4*D*). Among the excitatory responding neurons, the proportion of Type E1 (Fig. 2*D*) among all the responsive neurons was larger in the posterior part (Fig. 4*E*). These trends remained even after putative interneurons were excluded based on spike waveforms (Extended Data Fig. 4-1, Extended Data Table 1-1). Collectively, our anatomical and electrophysiological results suggest that the anterior and posterior parts of the motor cortex are differentially involved in USVs.

10.1523/ENEURO.0452-23.2024.f4-1 Figure 4-1 **Comparison of USV-response among putative pyramidal (projection) neurons.** **A**: Classification of neuron types based on spike width (trough-to-peak duration, inset). A group of neurons showing narrow (dark gray) and wide (light gray) spikes were separated using k-means clustering (k = 2) and estimated as putative interneuron (pIN) and putative pyramidal neurons (pPN), respectively, according to a previous study (Barthó et al., 2004). **B-F**: Same comparison as Fig. 4B-F but focusing on putative pyramidal neurons. ***, p < 0.001, **, p < 0.01, ^+^, p < 0.1, Fisher’s exact test with Bonferroni correction. The corresponding numbers of neurons of each type in each area are listed in Table 1-1. Download Figure 4-1, TIF file.

**Table 1.** Number of neurons in each response type in each area, corresponding to Figure 4

		E1	E2	I1	I2	I3	I4	O	NR	Total
M1	A	36	29	54	35	33	22	62	584	855
	P	18	10	14	8	4	3	16	193	266
M2	A	46	45	51	22	29	11	48	485	737
	P	13	3	11	2	0	1	6	132	168
Total		113	87	130	67	66	37	132	1,394	2,026

NR, nonresponsive neurons.

10.1523/ENEURO.0452-23.2024.t1-1 Table 1-1 Number of putative pyramidal neurons in each response type in each area, corresponding to Fig. 4-1. Download Table 1-1, DOC file.

**Table 2.** Statistical table

	Data structure	Type of test	Power
a	Normal distribution	Paired *t* test	95% CI of mean difference: [−32.1, 6.6]
b	-	Fisher's exact test	95% CI of odds ratio: [1.28, 2.84]
c	-	Fisher's exact test	95% CI of odds ratio: [0.29, 0.88]
d	-	Fisher's exact test	95% CI of odds ratio: [1.02, 2.91]
e	-	Fisher's exact test	95% CI of odds ratio: [0.25, 0.89]
f	-	Fisher's exact test	95% CI of odds ratio: [0.19, 0.84]
g	-	Fisher's exact test	95% CI of odds ratio: [1.20, 2.54]
h	-	Fisher's exact test	95% CI of odds ratio: [1.42, 3.41]
i	-	Fisher's exact test	95% CI of odds ratio: [0.16, 0.97]
j	-	Permutation *t* test	95% CI of median difference: [−0.0242, −0.0012]
k	-	Permutation *t* test	95% CI of median difference: [−0.0496, −0.0061]

### PAG-projecting neurons in the posterior part respond excitatorily to USVs

**Figure 5.**
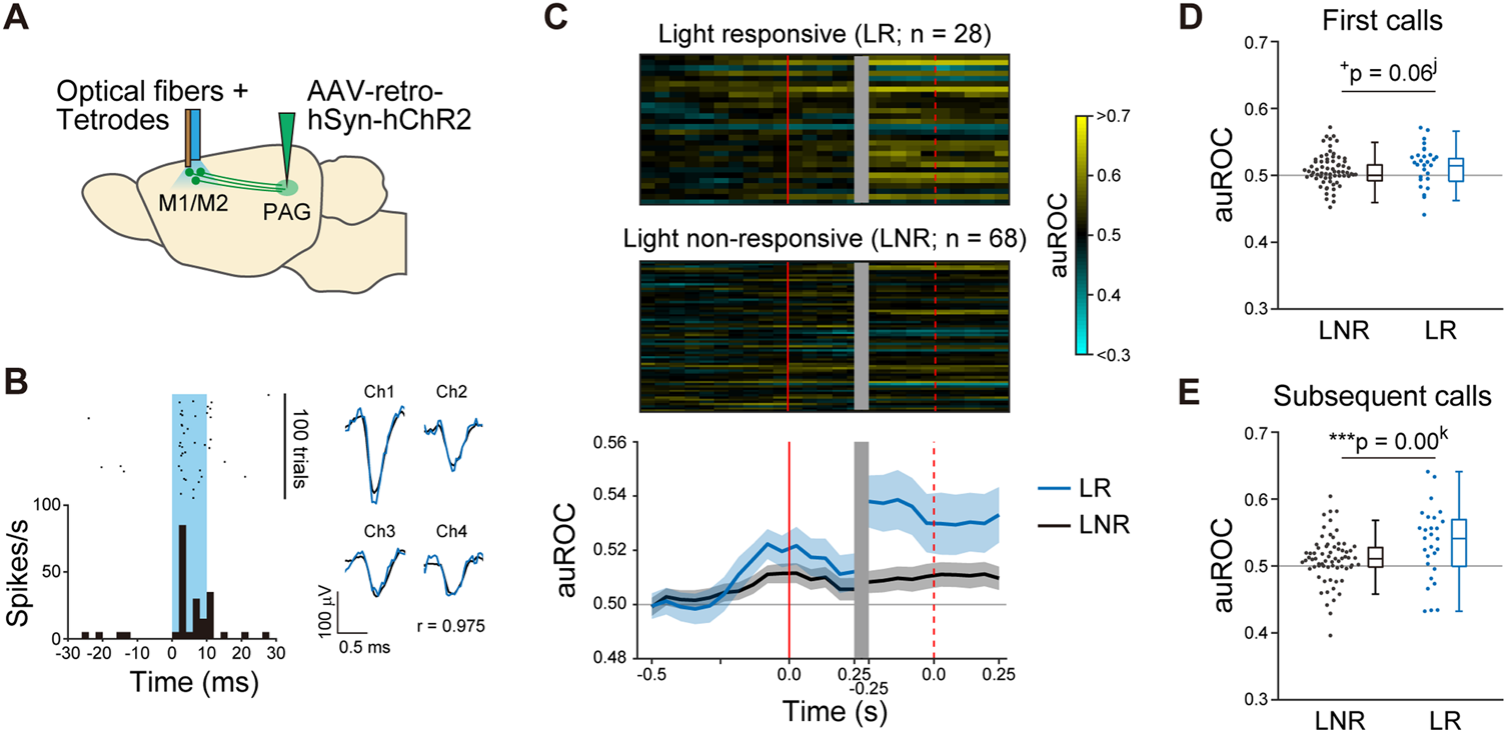
Optogenetically identified PAG projection neurons responded predominantly excitatorily to USVs. ***A***, Schematic of the optogenetic tagging experiment. ***B***, Left, Response to a 10 ms light pulse (light-blue shaded area) of a light-responsive neuron. Right, Averaged spike waveforms of the same neuron recorded during the light pulse (blue) and other periods (black). Ch 1–4 represent four channels of the tetrode. *r*, Pearson's correlation coefficient between the waveforms. ***C***, Responses of all light-responsive and nonresponsive neurons around the USVs (top) and their average (bottom). ***D***, ***E***, Comparison of mean auROC value in ±0.25 s around the first calls (***D***) and the subsequent calls (***E***) between light-responsive and nonresponsive neurons. ***p < 0.001, ^+^*p* < 0.05, permutation *t* test for median difference with Bonferroni’s correction (repetition, 2). ^j,k^Details of the statistical tests are shown in Table 2.

These results show that the distributions of PAG-projecting neurons and neuron types, based on response patterns to USVs, differ in the anterior and posterior parts of the motor cortex. To examine whether the PAG-projecting neurons in the motor cortex included those that responded to USVs, we recorded and analyzed the responses of these neurons to USVs using optogenetic tagging (Kvitsiani et al., 2013; Eshel et al., 2015; *n* = 3 rats; Fig. 5*A*,*B*). In this experiment, we focused on recordings from the posterior part, which are more likely to be directly related to USVs, since this part overlaps with the neck region of the motor cortex (Neafsey et al., 1986) and contains neurons projecting to the brainstem area that controls the laryngeal muscles (Arriaga et al., 2012). We recorded 28 light-responsive (LR) neurons and compared their responses to the USVs with those of 68 light-nonresponsive (LNR) neurons recorded from the same tetrodes. The anterior–posterior positions of these LR and LNR neurons were 0.85 ± 0.14 mm and 0.55 ± 0.09 mm, respectively (mean ± SEM; range, −0.1 to 2.0 mm). We found that the average response to USVs was significantly larger in LR neurons than that in LNR neurons (Fig. 5*C–E*). In addition, average response to the subsequent calls were significantly larger than the first calls in LR neurons (*p* = 0.0088; 95% CI of median difference = [0.0013, 0.0332]; permutation *t* test), but not in LNR neurons (*p* = 0.93; permutation *t* test). These results suggest that PAG-projecting neurons in the posterior part of the motor cortex predominantly show sustained excitatory response during USVs.

## Discussion

In this study, we recorded and analyzed neural responses to USVs across the motor cortex in rats (Fig. 2). We also investigated the projections of neurons in the motor cortex to the PAG and found two clusters of projecting neurons in the anterior and posterior parts of the motor cortex (Fig. 3). Interestingly, the USV response patterns of neurons differed between the anterior and posterior regions (Fig. 4). Finally, using optogenetic tagging, we recorded the USV responses of PAG-projecting neurons in the posterior part of the motor cortex and found that they showed predominantly excitatory responses during USVs (Fig. 5). These results indicate that the rat motor cortex is actively involved in the generation of USVs at neuronal and circuit levels.

We investigated the neurons projecting to the ventral and lateral PAG, which is reported to be an important region for courtship USVs in mice (Tschida et al., 2019) and 50 kHz USVs, which are associated with positive emotional states (Burgdorf et al., 2007; Brudzynski, 2009), and play behavior in rats (Gloveli et al., 2023). The female-induced USVs observed in this study were also categorized as 50 kHz USVs. We found that the distribution of neurons in the motor cortex projecting to the area of PAG consisted of two clusters located in the anterior and posterior parts (Fig. 3). Neurons in the anterior cluster were mostly found in M2 and continued to the medial prefrontal cortex (mPFC), which consists of the cingulate, prelimbic, and infralimbic cortices. Previous studies have reported that the activation and suppression of mPFC elicits and reduces USVs, respectively (Burgdorf et al., 2007; Bennett et al., 2019; Gan-Or and London, 2023), and that rats self-stimulate mPFC when the stimulation is conditioned with operant behavior (Olds, 1977; Burgdorf et al., 2007). Since the mPFC is also a critical area for social behaviors, such as social motivation, social recognition, and dominance hierarchy (Bicks et al., 2015), the anterior cluster may contribute to triggering USVs in appropriate social/affective contexts. In contrast, the posterior cluster extended across M1 and M2 (Fig. 3). The posterior part of the motor cortex overlaps with the area where microstimulation elicits neck movements (Neafsey et al., 1986). It has also been shown in mice that neurons in this area project their axons to the brainstem, which controls the laryngeal muscles (Arriaga et al., 2012). Laryngeal movements are shown to be critical for generating USVs in mice (Mahrt et al., 2016), and lesions in the posterior part of M1 increase deviation of pitch distribution (Arriaga et al., 2012). Thus, the posterior cluster identified in this study may contribute to motor control of USVs. Since the neurons located in the PAG and adjacent brainstem regions are implicated in various functions other than vocalization, such as defensive behavior, nociception, and autonomic regulation (Behbehani, 1995; Lefler et al., 2020; Nguyen et al., 2023), it will be of great interest to examine the effects of artificial suppression or activation of these clusters to clarify their role in USVs.

Several neurons in the posterior part of the motor cortex and PAG-projecting neurons in the posterior region showed excitatory modulation during USVs (Figs. 4, 5). Interestingly, previous studies in mice have suggested that excitatory inputs from the motor cortex to GABAergic interneurons in the auditory cortex are involved in suppressing the auditory response to self-generated sounds (Nelson et al., 2013; Schneider et al., 2014, 2018). Moreover, a fraction of these auditory cortex-projecting M2 neurons also branch their axons to the PAG and brainstem nuclei that are involved in vocalization (Nelson et al., 2013). These neurons could be involved in selective suppression of auditory responses to own vocalizations that are proposed to be essential for vocal communication to discriminate vocalizations of the self and others and maintain sensitivity to external sounds (Schneider and Mooney, 2018). Similar patterns of response suppression to the rat's own vocalizations have been found in the auditory cortex and amygdala (Rao et al., 2014; Matsumoto et al., 2016). Therefore, it is feasible that the excitatory response in the posterior parts may contribute to feedforward inhibition of reafferent sound to achieve normal communication, as well as the precise motor control (Arriaga et al., 2012).

This study had a few limitations that require further investigation. First, it remains unclear which aspects of vocal behaviors each responsive neuron represents, as vocalization patterns of 50 kHz USVs are highly variable (Wright et al., 2010) and often accompanied by bodily movements (Matsumoto et al., 2016). To further elucidate processing in the motor cortex, long-term stable recording of the same neurons and analysis using regression analysis (Engelhard et al., 2019) may help disentangle the neural representations of different aspects of USVs. Second, we did not examine whether the same circuits or neurons are involved in other types of vocalizations, such as 22 kHz USVs (Brudzynski, 2009) or audible vocalizations (Jourdan et al., 1995), both of which are often associated with a negative affective state. Distinct circuits may control different types of vocalizations, as a previous study in monkeys reported that the activation of different parts of PAG induced different types of vocalizations with different social meanings (Dujardin and Jürgens, 2005). Third, most of the lesion, functional suppression, and anatomical tracing experiments for investigating the role of the cerebral cortex on USVs (Arriaga et al., 2012; Nelson et al., 2013; Hammerschmidt et al., 2015; Ivanenko et al., 2020; Gan-Or and London, 2023) have been conducted with mice. For comprehensive understanding of the role of the motor cortex in controlling USV in rodents, similar studies using rats are necessary since it has been shown that there are slight differences between the two species in the organization of the motor cortex (Tennant et al., 2011) and several characteristics of USVs (Portfors, 2007).

In conclusion, we examined the involvement of the motor cortex in USVs at neuronal and circuit levels in the present study. We showed that neurons in M1 and M2 that respond to USVs can be divided into two clusters along the anterior–posterior axis. The cluster in the posterior area contains neurons projecting to the PAG, which may be involved in precise motor control of USVs, presumably based on auditory feedback of their own USVs and/or in auditory processing to suppress responses to their own USV sounds. Future studies investigating the interaction of the motor cortex circuits with other vocal production circuits, as well as with the auditory system, will strengthen and expand the usefulness of USVs as experimental models for investigating various neural mechanisms and disorders.

## Synthesis

Reviewing Editor: Nathalie Ginovart, University of Geneva Decisions are customarily a result of the Reviewing Editor and the peer reviewers coming together and discussing their recommendations until a consensus is reached. When revisions are invited, a fact-based synthesis statement explaining their decision and outlining what is needed to prepare a revision will be listed below. The following reviewer(s) agreed to reveal their identity: Jeffrey Burgdorf, Michael Brecht.

Both reviewers agreed that this is a good manuscript that provides new and important insights into the neuronal projection from the motor cortex to the PAG that that underlies the generation of ultrasonic vocalizations at the individual neuron level. Reviewer 2 highlighted some minor comments that need to be addressed before the paper can be accepted.

Please find the reviewer's comments below:

Reviewer 1:

The target article uses a state-of-the-art method to determine the functional neuronal circuit from the motor cortex to the PAG that controls the generation of ultrasonic vocalizations. The combination of single unit recording and optogenetics determined a role for excitatory neurons projecting to the PAG for the generation or fine motor modulation of ultrasonic vocalizations. This paper represents a major step forward in understanding the neuronal circuit of ultrasonic calls.

Reviewer 2:

In their brief paper 'Characterization of ultrasonic-vocalization-modulated-neurons in rat motor cortex based on their activity modulation and axonal projection to periaqueductal gray' the authors report on USV related responses in rat motor cortex. As far as I can see this is an important contribution, because (i) there is only limited information available on USV modulated cells in the rat motor cortex, (ii) there is only limited information available on the projection from rat motor cortex to the PAG. (iii) the experiments reported here are not trivial and appear to have been carefully done. With all that I support this paper and have only a set of minor suggestions.

1. The authors refer in their abstract to the hypothesis that rat motor cortex precisely controls USVs. As far as I am concerned there is no such hypothesis and I'd emphasize that the role of M1 in eat call control is rather unclear. Are there vocalization deficits in rats after M1 lesions or after decortication?

2. It would be great if the authors could provide a bit more info on the relationship of call types and neural discharges.

3. In the anterior part of motor cortex the PAG back-labelling appears to very sharply delineate the M1/ M2 border. Is that so and if yes please communicate this finding more clearly.

4. From Figure 5 C it appears to me that the big difference in the call responses between PAG-projecting and PAG-non-projecting cells lies the evolution of their activity in the call bout, where the PAG-projecting cells a much more sustained activity. Is that so and if yes is this effect statistically significant? If yes please report this finding in the abstract.

## Author Response

We wish to express our appreciation to the reviewers for their support of the significance of our paper.

We also appreciate the insightful comments which have helped us to improve the paper. The responses to each of the comments are as follows.

Reviewer 2 Comment 1: The authors refer in their abstract to the hypothesis that rat motor cortex precisely controls USVs. As far as I am concerned there is no such hypothesis and I'd emphasize that the role of M1 in rat call control is rather unclear. Are there vocalization deficits in rats after M1 lesions or after decortication? According to the reviewer's comment, we added the following text in Discussion (Lines 339-345) to clarify the limitation:

Third, most of the lesion, functional suppression, and anatomical tracing experiments for investigating the role of the cerebral cortex on USVs (Arriaga et al., 2012; Nelson et al., 2013; Hammerschmidt et al., 2015; Ivanenko et al., 2020; Gan-Or and London, 2023) have been conducted with mice. For comprehensive understanding of the role of the motor cortex in controlling USV in rodents, similar studies using rats are necessary since it has been shown that there are slight differences between the two species in the organization of the motor cortex (Tennant et al., 2011), and several characteristics of USVs (Portfors, 2007).

We also added the missing description about the species used in the previous studies in Introduction and Discussion, as follows.

Lines 48-55, in Introduction:

This may be partly due to the disputed role of cortical control in USVs, particularly in mice. A previous study showed that seemingly normal USVs were observed in mice lacking majority of the cortex, suggesting that cortical structures may not be critical for controlling USVs (Hammerschmidt et al., 2015). However, several anatomical tracing and immediate-early-gene-expression studies in mice have shown that the motor cortex projects to the PAG and nucleus ambiguus, a brainstem nucleus controlling the vocal muscles, and that it has a reciprocal connection with the auditory cortex (Arriaga et al., 2012; Nelson et al., 2013).

Lines 304-308, in Discussion:

It has also been shown in mice that neurons in this area project their axons to the brainstem, which controls the laryngeal muscles (Arriaga et al., 2012). Laryngeal movements are shown to be critical for generating USVs in mice (Mahrt et al., 2016), and lesions in the posterior part of M1 increase deviation of pitch distribution (Arriaga et al., 2012).

Comment 2: It would be great if the authors could provide a bit more info on the relationship of call types and neural discharges.

According to the reviewer's comment, we analyzed the relationship of call types and neural discharges and added the following texts in Methods and Results sections and Fig. 2-3.

Lines 182-187, in Methods:

To investigate the relationship between neural response and call types, we classified USVs into two types, i.e., flat (frequency range of the call < 15 kHz) and frequency modulated (frequency range of the call > 15 kHz) calls according to previous studies (Matsumoto et al., 2016; Simola and Granon, 2019). Then, significant discriminations of call types were determined using Wilcoxon rank sum test (p < 0.05) comparing neural response around the two types of calls (-0.25 to 0.25 s around the first call onsets).

Lines 225-227, in Results:

Among 632 responsive neurons, 48 (7.6 %) neurons were differently responded depending on call types (Fig. 2-3).

Comment 3: In the anterior part of motor cortex the PAG back-labelling appears to very sharply delineate the M1/ M2 border. Is that so and if yes please communicate this finding more clearly.

Thank you very much for the intriguing comment. We agree that there seems to be a sharp contrast in the number of labeled neurons between M1 and M2 in the anterior part. We modified the corresponding description in Results (Lines 242-245) accordingly as follows:

Interestingly, in the anterior part, a cluster of labeled neurons was observed continuously from the M2 to the cingulate, prelimbic, and infralimbic cortices while much fewer labeled neurons were found in M1 (Fig. 3C, Fig. 3-1).

Comment 4: From Figure 5 C it appears to me that the big difference in the call responses between PAG-projecting and PAG-non-projecting cells lies the evolution of their activity in the call bout, where the PAG-projecting cells a much more sustained activity. Is that so and if yes is this effect statistically significant? If yes please report this finding in the abstract.

According to the reviewer's comment, we added the following text in Results (Lines 273-278):

In addition, average response to the subsequent calls were significantly larger than the first calls in LR neurons (p = 0.0088, 95% CI of median difference = [0.0013, 0.0332], Permutation t-test), but not in LNR neurons (p = 0.93, Permutation t-test). These results suggest that PAG-projecting neurons in the posterior part of the motor cortex predominantly show sustained excitatory response during USVs.

We also changed Abstract as follows (Lines 15-17):

Furthermore, using optogenetic tagging, we recorded the USV modulation of PAG-projecting neurons in the posterior part of the motor cortex and found that they showed predominantly sustained excitatory responses during USVs.

In addition to the above revisions for responding to the reviewer's comments, we found and corrected a few small mistakes as follows:

Line 36: Simola et al., and Granon, 2019 Lines 177-180: First, the z-scores of four-dimensional features of the responsive neurons were mapped onto a two-dimensional plane using t-distributed stochastic neighbor embedding (t-SNE; Fig. 2-21A).

A watershed transform was then applied to a Gaussian-smoothed density of neurons to segment the space (Fig. 2-21B, C).
