# Supplementary figures and images for: Characterization of Ultrasonic Vocalization-Modulated Neurons in Rat Motor Cortex Based on Their Activity Modulation and Axonal Projection to the Periaqueductal Gray
Source: eNeuro. 2024 Mar 29;11(4):ENEURO.0452-23.2024. doi: 10.1523/ENEURO.0452-23.2024 (PMC10988357; doi:10.1523/ENEURO.0452-23.2024)

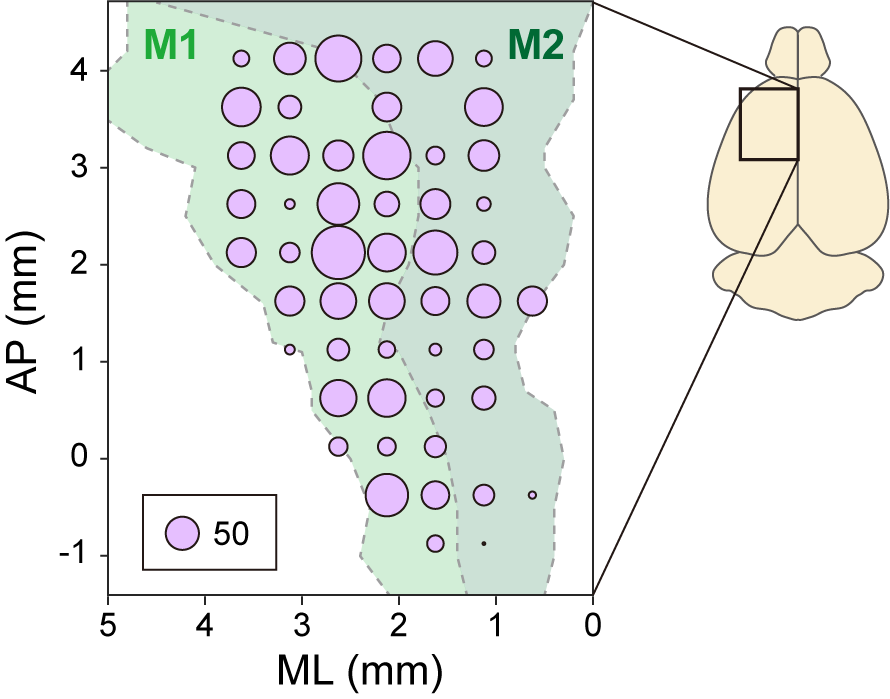

Supplement: Figure 2-1 — Number of recorded neurons across the motor cortex. Number of neurons recorded around each AP and ML coordinate is shown in the bubble chart. Shaded areas correspond to M1 and M2 on the brain surface, plotted according to the rat brain atlas (Paxinos and Watson, 2007). Download Figure 2-1, TIF file. [file eneuro-11-ENEURO.0452-23.2024-s002.tif]

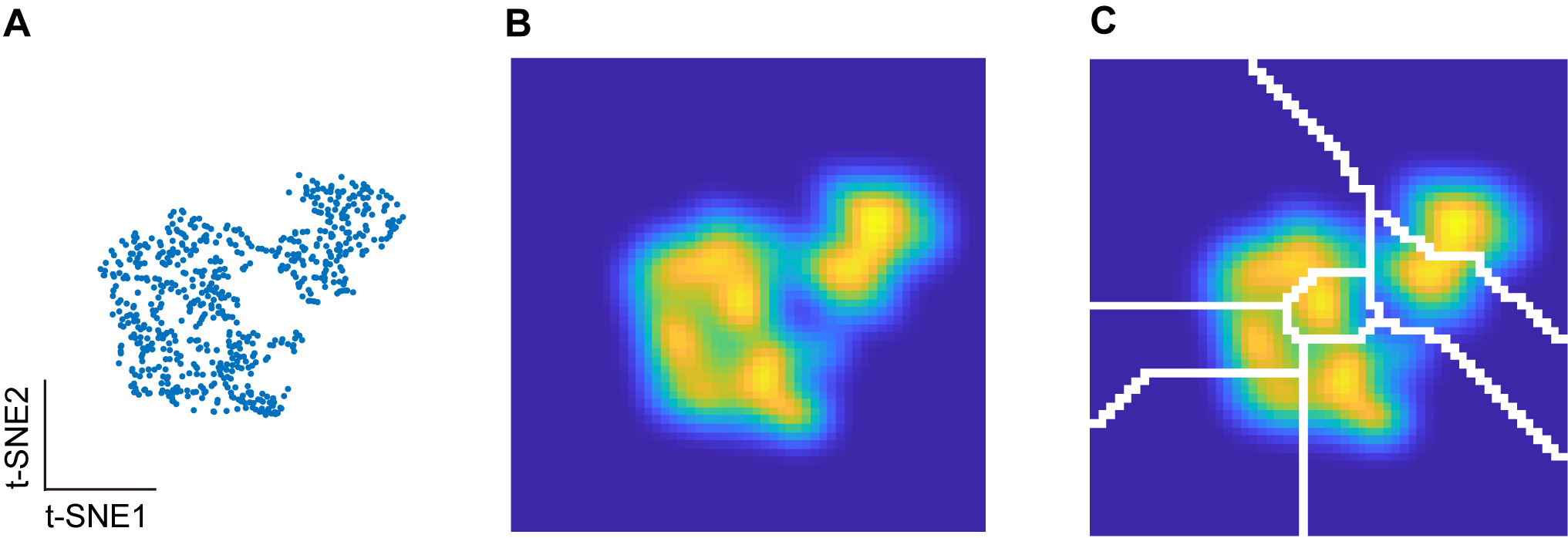

Supplement: Figure 2-2 — Unsupervised classification using watershed algorithm. From the two-dimensional plot obtained using t-SNE (A), a 2D histogram was generated and smoothed using a Gaussian filter (B). Individual peaks were isolated via watershed transform (C). Download Figure 2-2, TIF file. [file eneuro-11-ENEURO.0452-23.2024-s003.tif]

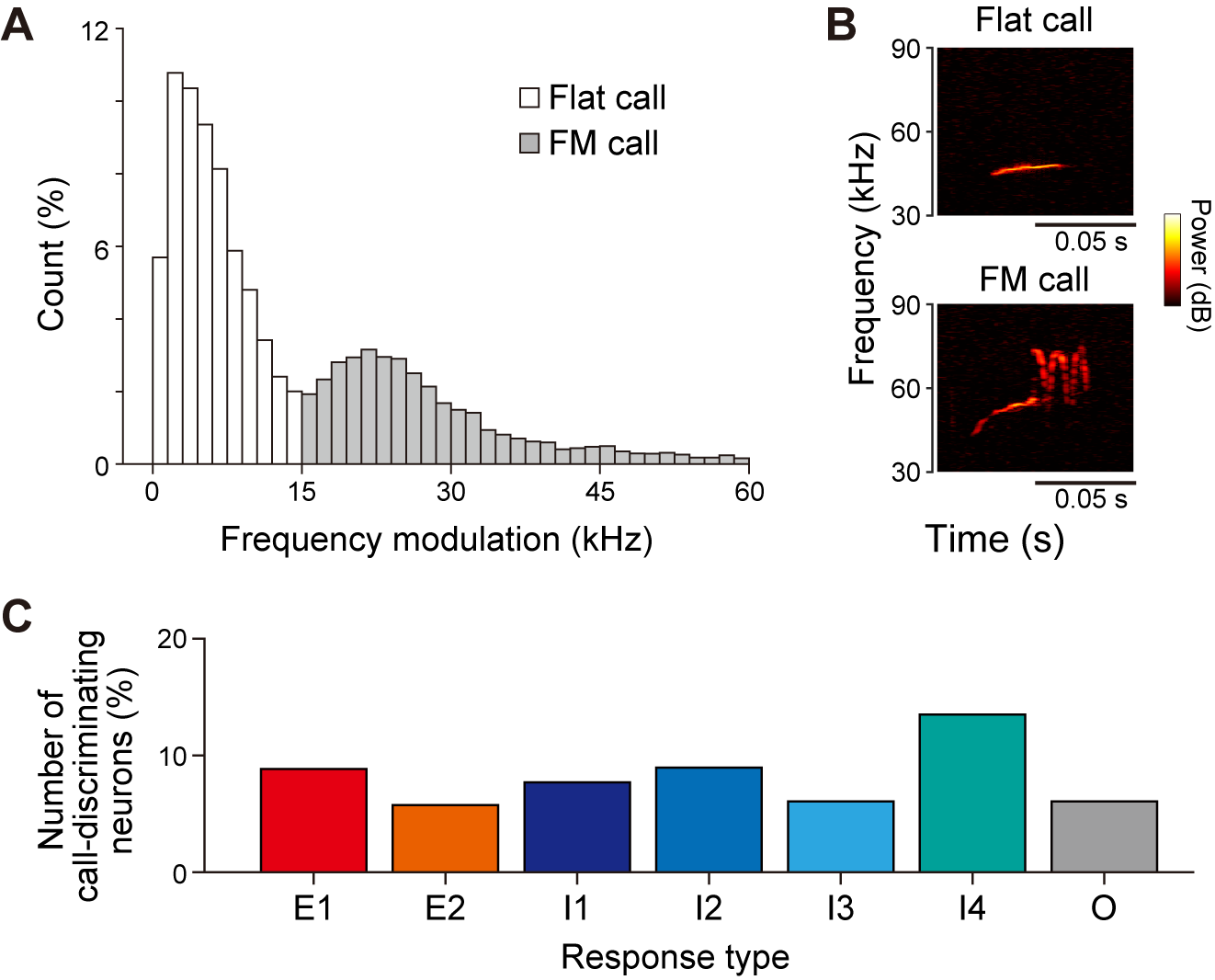

Supplement: Figure 2-3 — Count of call-discriminating neurons. A: Distribution of frequency ranges (max frequency - min frequency) of all recorded USVs (n = 20,195). The USVs were classified into flat (<15 kHz), and frequency modulated (FM; > 15 kHz) calls based on the values. B: Example spectrograms of the two call types. C: Percentage of call-discriminating neurons among each type of USV responsive neurons. Download Figure 2-3, TIF file. [file eneuro-11-ENEURO.0452-23.2024-s004.tif]

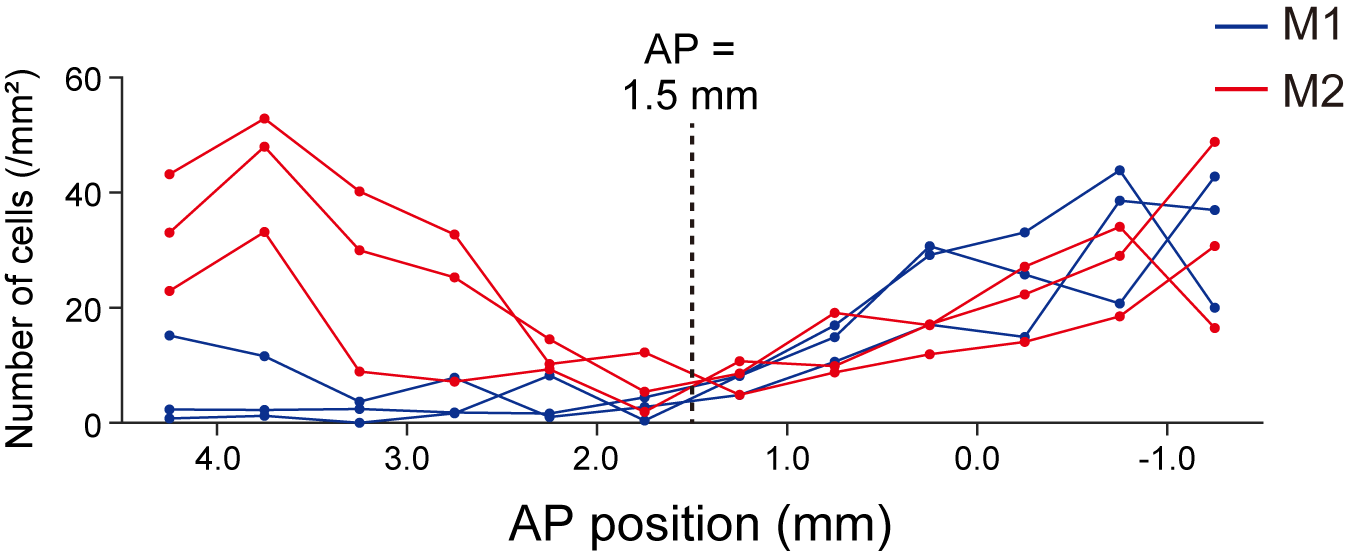

Supplement: Figure 3-1 — Distribution of the retrogradely-labeled cells in AP axis. Densities at different AP levels for each animal are shown as single lines (blue, M1; red, M2). We divided the anterior and posterior part by 1.5 mm (dotted line) for the analyses in Fig. 3 and 4. Download Figure 3-1, TIF file. [file eneuro-11-ENEURO.0452-23.2024-s005.tif]

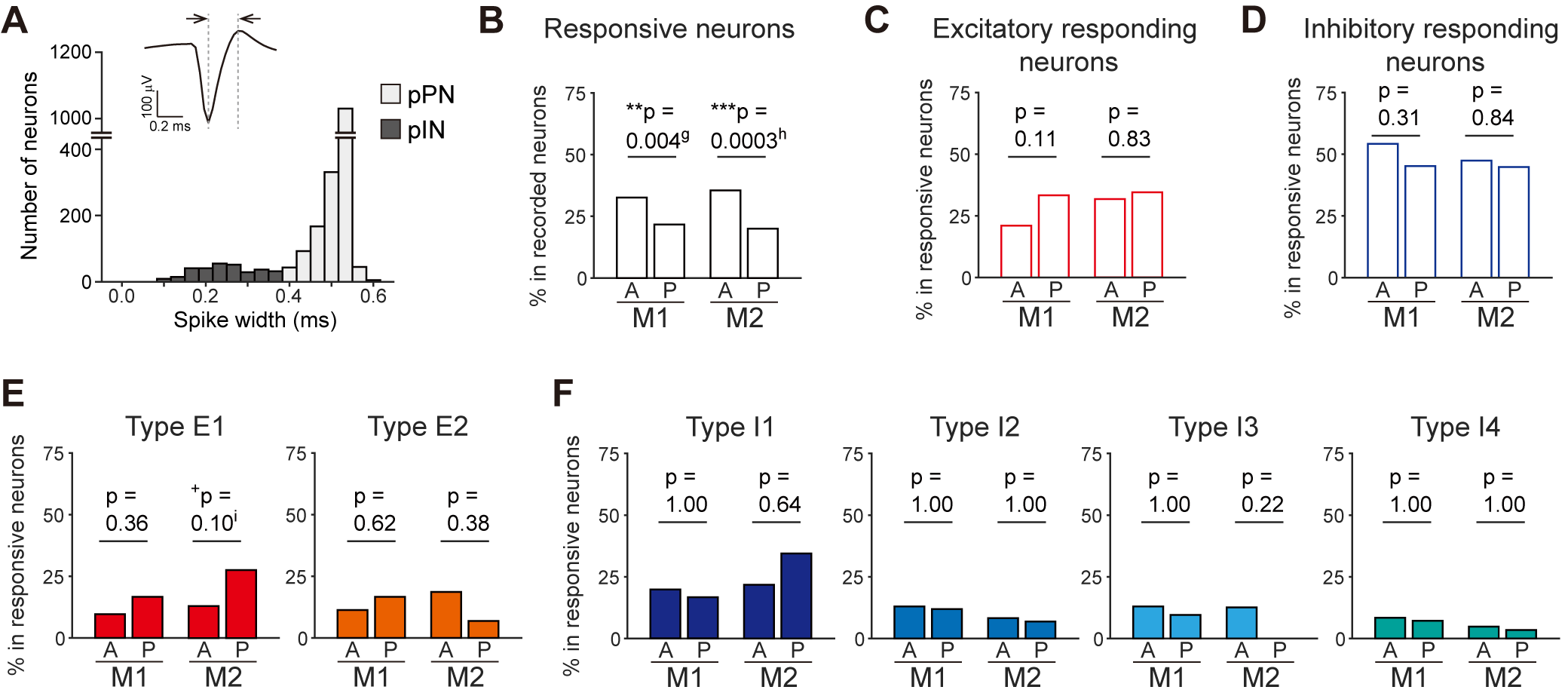

Supplement: Figure 4-1 — Comparison of USV-response among putative pyramidal (projection) neurons. A: Classification of neuron types based on spike width (trough-to-peak duration, inset). A group of neurons showing narrow (dark gray) and wide (light gray) spikes were separated using k-means clustering (k = 2) and estimated as putative interneuron (pIN) and putative pyramidal neurons (pPN), respectively, according to a previous study (Barthó et al., 2004). B-F: Same comparison as Fig. 4B-F but focusing on putative pyramidal neurons. ***, p < 0.001, **, p < 0.01, +, p < 0.1, Fisher’s exact test with Bonferroni correction. The corresponding numbers of neurons of each type in each area are listed in Table 1-1. Download Figure 4-1, TIF file. [file eneuro-11-ENEURO.0452-23.2024-s006.tif]
