# Supplementary material for: Characterization of Ultrasonic Vocalization-Modulated Neurons in Rat Motor Cortex Based on Their Activity Modulation and Axonal Projection to the Periaqueductal Gray
Source: eNeuro. 2024 Mar 29;11(4):ENEURO.0452-23.2024. doi: 10.1523/ENEURO.0452-23.2024 (PMC10988357; doi:10.1523/ENEURO.0452-23.2024)
Supplement: Table 1-1 — Number of putative pyramidal neurons in each response type in each area, corresponding to Fig. 4-1. Download Table 1-1, DOC file. [file eneuro-11-ENEURO.0452-23.2024-s007.doc]

|  |  | E1 | E2 | I1 | I2 | I3 | I4 | O | NR | Total |
| --- | --- | --- | --- | --- | --- | --- | --- | --- | --- | --- |
| M1 | A | 23 | 27 | 47 | 31 | 31 | 20 | 59 | 491 | 729 |
|  | P | 7 | 7 | 7 | 5 | 4 | 3 | 9 | 151 | 193 |
| M2 | A | 30 | 43 | 50 | 19 | 29 | 11 | 48 | 418 | 648 |
|  | P | 8 | 2 | 10 | 2 | 0 | 1 | 6 | 116 | 145 |
| Total | | 68 | 79 | 114 | 57 | 64 | 35 | 122 | 1176 | 1715 |

**(Extended data) Table 1-1. Number of putative pyramidal neurons in each response type in each area, corresponding to Fig. 4-1.**

NR, non-responsive neurons.
